# Choosing the most appropriate cut-point for continuous variables

**DOI:** 10.1590/0100-6991e-20223346-en

**Published:** 2022-07-19

**Authors:** FRANCISCO TUSTUMI

**Affiliations:** 1 - Universidade de São Paulo, Gastroenterologia - São Paulo - SP - Brasil; 2 - Hospital Israelita Albert Einstein, Cirurgia - São Paulo - SP - Brasil; 3 - Centro Universitário Lusíada, Departamento de Medicina Baseada em Evidências - Santos - SP - Brasil

Generically, variables can be classified as categorical or continuous. Categorical variables are a finite number of categories, such as gender and oncologic stage (I-IV). Continuous variables are numeric variables with infinite possible values between any two values. 

Several biomarkers routinely used in clinical practice are continuous, such as red and white blood cells count, Ki-67 expression, body mass index (BMI), carcinoembryonic antigen (CEA) level, and SUVmax uptake, among others.

Cut-points can be used in continuous variables to “discretize” a biomarker into different categories, providing benchmarks by which individuals will be classified in a group. The great advantage of applying cut-points is that threshold parameters ease decision-making. In clinical practice, clinicians need to know what group their patients are located to establish the proper diagnosis, treatment, or prognostic. “Is this Ki-67 value a sign of worry or not?”; “Does this BMI demand a bariatric procedure or not?”; “Is this SUVmax value positive or negative?”; “What should I do with this biomarker?”.

**Figure 1:**
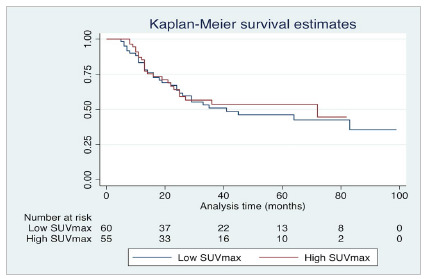
Survival analysis comparing groups of low and high SUVmax for esophageal cancer. A cut-point of 13.25 was determined. Analysis suggested that SUVmax was not a prognostic variable (p-value for log-rank: 0.699). Graph designed in Stata 16.0 statistical software (StataCorp LLC, Texas, USA).

**Figure 2:**
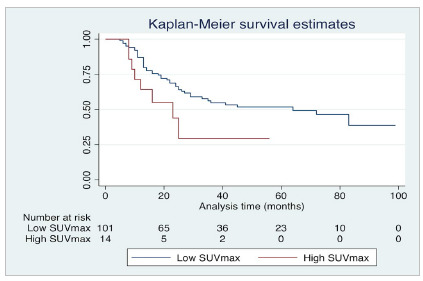
Survival analysis comparing groups of low and high SUVmax for esophageal cancer. A cut-point of 20 was determined. Analysis suggested that high SUVmax was associated with a poorer prognosis (p-value for log-rank: 0.045). Graph designed in Stata 16.0 statistical software (StataCorp LLC, Texas, USA).

Several methods have been proposed to find the best cut-point for each study. The authors and scientific paper readers underestimate the value of the methods for deciding on any cut-point. Papers often do not detail what method (or why a particular method) was chosen for picking up a certain cut-point for continuous variables. Actually, changing the cut-point can dramatically impact the studies’ conclusions. Depending on the cut-point chosen, the p-value can vary, and a null hypothesis can be accepted or rejected. In this sense, the researcher could easily bring evidence of both positive and null associations between two variables only by changing the cut-point set! In Figures 1 and 2, the value of the relation of the SUVmax in PET-CT with survival in esophageal cancer was investigated. By using a 13.25 value cut-point for SUVmax (Figure 1), the p-value for log-rank was 0.699, and we would demonstrate that there is no difference in survival rate between low (<13.25) and high (≥13.25) SUVmax. However, if we opt to change the cut-point to 20 (Figure 2), we would show evidence of a difference between survival curves, with a p-value of 0.045.

Probably, the simplest way to determine the cut-point is by the median value. The median value dichotomizes the biomarker, guaranteeing an equal sample size for both groups. 

In contrast, outcome-based methods allow finding an “optimal” cut-point. Using statistical methods, researchers can choose the cut-point according to the optimal separation between groups concerning some outcome. 

The first thing researchers need to consider before choosing the cut-point is to clearly establish what they are investigating. The choice of the cut-point should be based on how the independent continuous variable “y” relates with the dependent variable “x”: as a binary or time-to-event outcome.

**Figure 3:**
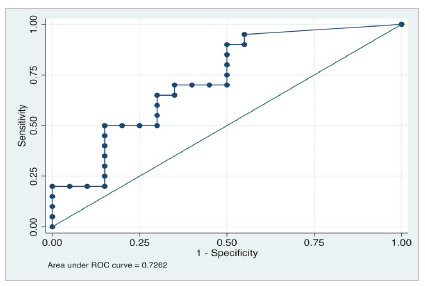
ROC curve. Graph designed in Stata 16.0 statistical software (StataCorp LLC, Texas, USA).

**Figure 4:**
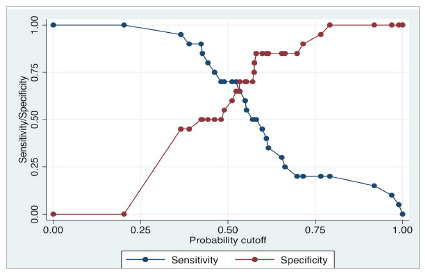
Relation of the sensitivity and specificity with the probability cutoff. Graph designed in Stata 16.0 statistical software (StataCorp LLC, Texas, USA).

For diagnostic studies, the most used resource for the continuous variable is the receiver operating characteristic (ROC) curves, evaluating a binary outcome: “The patient has the disease or not?”; “The test is positive or negative?”. This graph (Figure 3) shows the relation between the sensitivity and (1 - specificity), or the relationship between true and false positives. Each point in the graph shows the sensitivity and specificity relation according to a certain cut-point. The sensitivity and specificity in relation to the probability cutoff are demonstrated in Figure 4. In 1950, Youden^1^ first reported one of the strategies most used for ROC curves cut-points identification. The Youden index (J) maximizes the true positive and true negative rates. The equation is J = Sensitivity + Specificity - 1, which is applied for each point of the ROC curve. The maximum J is often used as a cut-point. Graphically, the J maximum point represents the maximum vertical distance between the 45-degree line and the point on the ROC curve.

However, any diagnostic investigation is based on the diagnostic performance parameters, such as specificity, sensitivity, likelihood ratio, and predictive values. The choice for the optimal cut-point ideally should be based on what one would expect for diagnostic test performance parameters for a particular disease. A high-risk or rapidly growing disease would demand tests with high sensitivity and high negative predictive value (diagnosing the majority of patients even at the expense of a high percentage of false positives). The test’s goal is to “ward off” a certain disease. The cut-point would be chosen as convenient according to the disease and located more on the right side of the ROC curve. For example, any certain biomarker expected to screen for colorectal cancer should have high sensitivity and high negative predictive value.

On the other hand, chronic disease or a disease that would demand a high-risk treatment should have high specificity and a high positive predictive value test. The purpose of the test would be “ratifying” a diagnostic suspicion. The choice biomarker cut-point would be chosen as convenient according to the disease and set more on the left of the ROC curve. 

Prognostic studies often evaluate time-to-event outcomes, such as survival rates. This type of evaluation brings up a new variable: the follow-up time. If a ROC curve is used for time-to-event outcomes, the heterogeneous length of follow-up due to the censored observations among patients is misinterpreted as homogeneous follow-up. For example, in survival analysis, patients that died at a 5-year follow-up would be evaluated as the same patients that died at a 1-year follow-up. That is completely inappropriate! 

Consequently, researchers frequently choose their cut-point according to the minimal p-value approach^2^. Researchers evaluate all possible cut-points and select the one that yields the smallest p-value between groups in survival analysis. However, this evaluation pattern is prone to the “looking for the pony bias”, also known as “data-dredging bias”. When data analysis is repeated several times until data can be presented as statistically significant, the risk of false positives grows (you will find the “p” you were looking for!). In addition, by choosing the minimal p-value, there is a risk of overestimating the effect size measure. 

**Figure 5:**
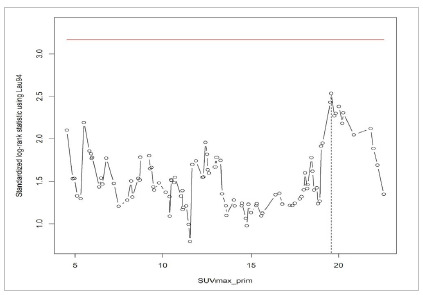
The maximally selected rank statistics according to Lausen ^3^ . Graph designed in R Core Team, 2016 statistical software (R Foundation for Statistical Computing, Vienna, Austria).

Lausen^3^ proposed a new form of cut-point determination, in which the follow-up time would be eventually incorporated for time-to-event outcomes. The maximally selected rank statistics divides the patients into two groups with the most significant statistics between each other. The aim is to find out the maximum of the standardized statistics of all possible cut-points, which can provide the best separation into two groups of survival (Figure 5). The exact conditional p-value can be estimated with the Monte-Carlo simulation. The authors also^2^ proposed a mathematical improvement, correcting the p-value by using a formula to minimize the type-I error. Other authors also proposed different methods for α-level adjustment^4^ ^,^ ^5^. Bonferroni^5^ suggested a simple correction by dividing the α-level by the number of candidate cut-points. 

However, it is essential to note that applying cut-points will always impose some limitations in their interpretation. The use of threshold supposes the existence of a straightforward step that apart “positive” and “negative”, “high-risk” and “low-risk”, when actually, continuous variables represent a broad spectrum of prognosis possibilities or even a wide range of diagnostic performance parameters possibilities.

Independently of the cut-point model chosen, researchers should apply different strategies to demonstrate the actual value of the cut-point models. Regression models should be used for prognostic studies, and sensitivity analysis should also be considered. Establishing the optimal cut-point in different independent datasets can also help minimize the risk bias and type-I error. Besides, scientific manuscripts readers should always have a critical mentality and have the capability to perceive if the cut-point used in certain papers was reasonable or could be providing biased information.
